# Antimicrobial Mechanism and Secondary Metabolite Profiles of Biocontrol Agent *Streptomyces lydicus* M01 Based on Ultra-High-Performance Liquid Chromatography Connected to a Quadrupole Time-of-Flight Mass Spectrometer Analysis and Genome Sequencing

**DOI:** 10.3389/fmicb.2022.908879

**Published:** 2022-05-31

**Authors:** Mingxuan Wang, Jing Li, Wenjie Cong, Jianguo Zhang

**Affiliations:** Institute of Food Science and Engineering, School of Health Science and Engineering, University of Shanghai for Science and Technology, Shanghai, China

**Keywords:** *Streptomyces lydicus*, antimicrobial mechanism, biopesticides, secondary metabolites, UPLC-Q-TOF-MS, genome sequencing

## Abstract

*Streptomyces lydicus* was used as biopesticide for crop protection in agriculture, however, the antimicrobial mechanism remains unclear and no systematic research on the secondary metabolites of *S. lydicus* has been reported. In this study, the extract of *S. lydicus* M01 culture was used to treat plant pathogen *Alternaria alternata* and morphological changes in the plasma membrane and cell wall of hyphae and conidia were observed. Fluorescence microscopy combined with different dyes showed that the accumulation of reactive oxygen species and cell death were also induced. To investigate the secondary metabolites in the culture filtrate, an online detection strategy of ultra-high-performance liquid chromatography connected to a quadrupole time-of-flight mass spectrometer (UPLC-Q-TOF-MS) was used for identification. The results revealed an excess of 120 metabolites, mainly consisted of fungicides, antibacterial agents, herbicides, insecticides, and plant growth regulators, such as IAA. Among which the five dominant components were oxadixyl, chloreturon, S-metolachlor, fentrazamide, and bucarpolate. On the other hand, the complete genome of *S. lydicus* M01 was sequenced and a number of key function gene clusters that contribute to the biosynthesis of active secondary metabolites were revealed. This is the first systematic characterization of *S. lydicus* secondary metabolites, and these results offer novel and valuable evidence for a comprehensive understanding of the biocontrol agent *S. lydicus* and its application in agriculture.

## Introduction

Sustainable agriculture attracts a growing demand of bio-based fertilizers composed of living microorganisms alternative to agro-chemicals (Kumawat et al., 2022). Biocontrol agents are microorganisms that suppress soilborne plant pathogens and promote plant growth. Many strains that have antagonistic activities were developed as commercial biocontrol agents to control plant diseases (Palaniyandi et al., 2013). Species from the *Streptomyces* genus exhibit marked biological activities against a wide range of phytopathogenic fungi and are considered as typical representatives of efficient biocontrol agents. They protect plants in direct and indirect ways; the direct effects might rely on the production of bioactive compounds that antagonize plant pathogen while indirect effects rely on induced systemic resistance (ISR) of plants (Raupach et al., 1996; Pieterse et al., 2014).

*Streptomyces* produce diverse natural products that accounts for approximately 10,000 compounds (Liu et al., 2012). The majority of antibiotics used in medicine and agriculture originated from *Streptomyces* bacteria. Their ability to produce various bioactive compounds by the biocontrol agents is desirable as it leads to suppression of diverse plant pathogens. For example, *Streptomyces aureofaciens* CMUAc130 has been reported to produce 5,7-dimethoxy-4-p-methoxylphenylcoumarin and 5,7-dimethoxy-4-phenylcoumarin, which can inhibit *Colletotrichum musae* and *Fusarium oxysporum*, the causative agents of anthracnose of banana and wilt of wheat (Taechowisan et al., 2005). *Streptomyces cavourensis* SY224 was found to produce a heat-stable antifungal compound, 2-furancarboxaldehyde that is antagonistic to *Colletotrichum gloeosporioides* which infects pepper plants (So Youn et al., 2012). *Streptomyces malaysiensis* produced a novel perhydrofuropyran C-nucleoside malayamycin that exhibited promising antifungal activity against *Stagonospora nodorum* (Berk) Castell & Germano, the cause of wheat glume blotch disease (Li et al., 2008), and an antifungal cyclic thiopeptide cyclothiazomycin B1 was isolated from the culture broth of *Streptomyces* sp. HA 125-40 that inhibited the growth of several plant pathogens (Mizuhara et al., 2011).

Another strain is the well-known *Streptomyces lydicus* WYEC108, which was found to antagonize against various fungal plant pathogens, including *Pythium ultimum* and *Rhizoctonia solani*, and promote the growth of pea plants (Crawford Don et al., 1993; Yuan and Crawford, 1995; Tokala Ranjeet et al., 2002). *S. lydicus* WYEC108 was developed and commercialized into biocontrol products Actinovate^®^ and Actino-Iron^®^ (Crawford et al., 2005). The antifungal ability of *S. lydicus* might be related to antibiosis, nutrient competition, and biosynthesis of degradative enzyme, although lytic enzymes, such as chitinases, were reported (Mahadevan and Crawford, 1997), its antimicrobial mechanism remains unclear and no detailed secondary metabolite profiles of *S. lydicus* was reported.

Recently, we isolated a new stain *S. lydicus* M01 that both strongly inhibit the leaf spot pathogen *Alternaria alternata* and significantly promoted the growth of cucumber. In pot experiments, pretreatment with *S. lydicus* M01 significantly reduced the disease incidence of foliar disease caused by *A. alternata* on cucumbers and the shoot length, root length, and fresh weight of the cucumber seedling were increased. In addition, *S. lydicus* M01 also exhibited growth promoting characteristics, such as phosphate solubilization, IAA secretion, siderophore, and ACC deaminase production (Wang et al., 2020). Thus, these beneficial activities, including regulation of soil microbial community, fight against pathogens and plant promotion effects make it a promising biological control agent. To further investigate its biological control and antimicrobial mechanism, in this study, the secondary metabolite profiles of *S. lydicus* M01 were analyzed using liquid chromatography connected to a quadrupole time-of-flight mass spectrometer (UPLC-Q-TOF-MS). On the other hand, the complete genome sequencing of *S. lydicus* M01 also revealed a number of key gene clusters that may contribute to the biosynthesis of active secondary metabolites.

## Materials and Methods

### Strains and Growth Conditions

*Streptomyces lydicus* M01 was isolated from a rhizosphere soil in Nanjing Laoshan Forest (Wang et al., 2020). The plant pathogen *A. alternata* (ACCC 36970) was purchased from the Agricultural Culture Collection of China. *S. lydicus* M01 was cultured on ISP-2 plate at 28°C for 14 days and was fermented in ptato dextrose broth (PDB) on a rotary shaker (200 rpm) for 4 days at 28°C. The plant pathogen *A. alternata* was cultured on potato dextrose agar (PDA) plates at 28°C for 4 days.

### Extraction of *Streptomyces lydicus* M01 Secondary Metabolites

Approximately 2 L of the *S. lydicus* M01 culture filtrate was collected from the PDB fermentation. The culture filtrate was extracted with an equal volume of ethyl acetate at 37°C with shaking at 120 rpm. The organic phase was separated from the liquid media using a separatory funnel. Then the organic layer was evaporated slowly under reduced pressure at 37°C to obtain an ointment crude extract. The concentrated extract was re-dissolved in 2 mL methanol. After filtration through a 0.22-mm sterile filter (Millipore, Bedford, MA, United States), the extracts were subjected to antifungal activity assay and UPLC-MS analysis.

### Effects of *Streptomyces lydicus* M01 Extracts on the Ultrastructure of *Alternaria alternata*

The hyphae and conidia of *A. alternata* were treated with the *S. lydicus* M01 extracts for 12 h. Scanning electron microscopy and transmission electron microscopy were used to determine the effects of *S. lydicus* M01 extracts on the hyphae and conidia at the ultrastructural level.

For scanning electron microscopy, after centrifugation, the hyphae and conidia were pre-fixed with 2.5% glutaraldehyde. The fixed cells were rinsed with 100 mM phosphate buffer for 10 min and post-fixed for 3 h in 1% osmium tetroxide solution, and then dehydrated using gradient ethanol. The samples were coated with gold and observed with a scanning electron microscope (SEM, S-3000N, Hitachi, Japan). For transmission electron microscopy, samples were embedded in Epon 812 resin and the embedded materials were sectioned using an ultramicrotome and examined with a transmission electron microscope (TEM, H-600, Hitachi, Japan).

### Viability Staining of *Streptomyces lydicus* M01 Extracts Treated *Alternaria alternata*

The fluorescein diacetate (FDA) stain and propidium iodide (PI) stain were used for cell viability assay (Jones and Senft, 1985), live cells with intact membranes show green fluorescence and cells with damaged membranes show red fluorescence. The hyphae and conidia of *A. alternata* were treated with *S. lydicus* M01 extracts for 12 h. After centrifugation, cells were resuspended in 20 mM phosphate buffer (pH 7.5) and stained with prepared FDA and PI, and incubated for 10 min in the dark at room temperature. Images were viewed using Olympus CX43 (Tokyo, Japan) microscope taken randomly from three independent experiments.

### Reactive Oxygen Species Detection

The intracellular reactive oxygen species (ROS) production was examined with dichlorodihydrofluorescein diacetate (DCFH-DA) (Sigma-Aldrich, Burlington, MA, United States) and fluorescence microscopy (Liu et al., 2010). The *A. alternata* hyphae and conidia were treated with *S. lydicus* M01 extracts for 6 h, centrifuged, and resuspended in 20 mM phosphate buffer (pH 7.5). The samples were then incubated with 20 μM DCFH-DA for 20 min at room temperature and viewed using Olympus CX43 microscope.

### UPLC-MS Analysis

The UPLC-MS analysis was performed on Acquity I-Class UPLC coupled with Xevo G2-XS QTof system equipped with electron spray ionization (ESI) ion source and UNIFI software (Waters, Milford, MA, United States). The extracts from *S. lydicus* M01 were separated on a Waters Acquity UPLC BEH C18 column (100 mm × 2.1 mm, 1.7 μm) maintained at 30°C. The mobile phase consisted of water (A) and acetonitrile (B) with an initial condition of 95% of mobile phase A and 5% of mobile phase B. The linear gradient elution was performed as follows: 0–10 min, 5% B; 10–15 min, 5–15% B; 15–45 min, 15% B. The flow rate was 0.4 ml/min. The column temperature was 30°C. Mass spectrometry was recorded using Xevo G2-XS QTOF equipped with an ESI source. The ionization mode was set in positive mode for identification of the *S. lydicus* M01 metabolites. The full MS scan was performed in the range m/z 100–1200 Da in-sensitivity mode with a scan time of 0.2 s. The capillary voltage was set to 2 kV, the cone voltage was 40 V. The cone gas flow rates and desolvation flow rates were 50 and 800 L/h, respectively. The collision energy was set at 35 eV and ramp high energy from 10 to 40 eV.

### Genome Sequencing and Metabolite Prediction of *Streptomyces lydicus* M01

*Streptomyces lydicus* M01 were cultured in the ISP-2 medium at 28°C for 48 h. The genomic DNA extraction was performed using Wizard^®^ Genomic DNA Purification Kit (Promega, Madison, MI, United States) according to manufacturer’s protocol. The complete genome sequencing and assembly were performed by the Majorbio Bio-Pharm Technology Co. Ltd., Shanghai, China. Genomic DNA was sequenced using a combination of PacBio RS II Single Molecule Real Time (SMRT) and Illumina sequencing platforms. The data generated from PacBio and Illumina platform were used for bioinformatics analysis using the online platform of Majorbio Cloud Platform.^1^ Glimmer (Delcher et al., 2007) was used for CDS prediction, tRNA-scan-SE (Borodovsky and McIninch, 1993) was used for tRNA prediction, and Barrnap was used for rRNA prediction. The predicted CDSs were annotated from NR, Swiss-Prot, Pfam, GO, COG, and KEGG databases. The biosynthetic gene clusters of secondary metabolites of *S. lydicus* M01 were predicted using the online antiSMASH v6.0.1 software (Blin et al., 2021).

## Results

### Microscopic and Ultrastructural Changes to *Alternaria alternata* Hyphae and Conidia Caused by *Streptomyces lydicus* M01 Extracts

**FIGURE 1:**
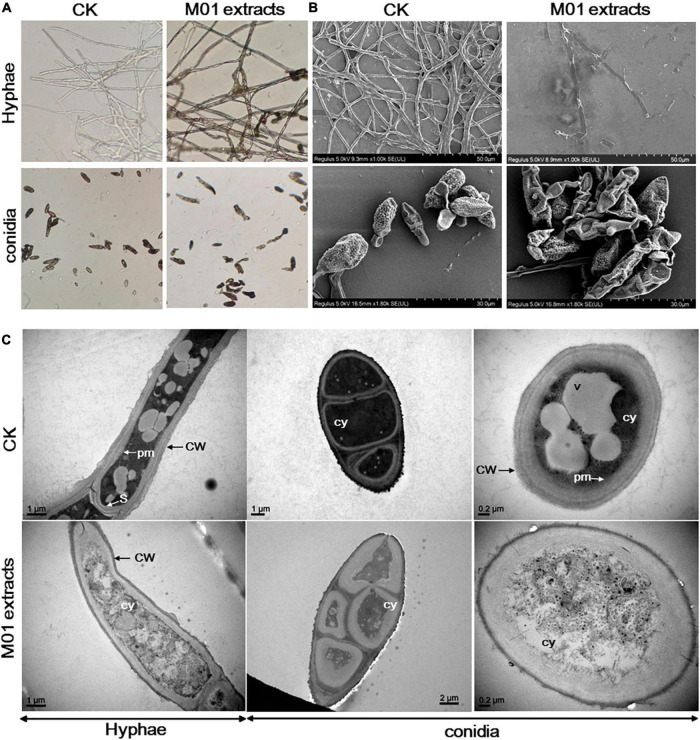
Effect of *Streptomyces lydicus* M01 extracts on the morphology of *Alternaria alternata* hyphae and conidia. **(A)** Effect of M01 extracts on the morphology of *A. alternata* hyphae and conidia observed with a light microscope. Ultrastructural effects of *S. lydicus* M01 extracts on *A. alternata* determined by scanning electron microscopy **(B)** and transmission electron microscopy **(C)**. CW, cell wall; pm, plasma membrane; S, septum; cy, cytoplasm. In control experiments (CK), *A. alternata* hyphae and conidia was treated with methanol.

To investigate the antimicrobial mechanism of *S. lydicus* M01, the leaf spot pathogen *A. alternata* was treated with *S. lydicus* M01 extracts. The morphological changes of fungal mycelia and spores were observed by light microscopy. In the control experiments, the untreated hyphae displayed regular morphology with smooth external surface and round apexes (Figure 1A). In the treatment group, deformed and wrinkled surface, melanization and curling of the hyphae were observed. The conidia treated with *S. lydicus* M01 extracts also appeared abnormal and shriveled. To further study the effects of *S. lydicus* M01 extracts on the ultrastructure of *A. alternata*, scanning electron microscopy and transmission electron microscopy analysis was conducted. The results of scanning electron microscopy showed that the untreated hyphae and conidia were intact, continuous, and plump. When treated with the *S. lydicus* M01 extracts, significant exterior damage of hyphae and conidia was observed, including irregular surface, fracture of hyphae, and shriveled conidial spores, indicating a leakage of cytoplasm out of the cells (Figure 1B). Under transmission electron microscopy, intact cell wall and well-defined plasma membrane with septum were observed. And vesicles were clearly visible in the cytoplasm of cells. After treatment with the *S. lydicus* M01 extracts, the cell walls of hyphae were found to be rough and uneven, whereas the plasma membrane became blurred and disappeared especially in the conidial spores observed (Figure 1C). Furthermore, cytoplasmic heterogeneity and disintegrated organelles were observed in the treated cells. These results revealed that the extracts of *S. lydicus* M01 may cause defects in cell wall integrity, cytoplasm extravasation, and cell lysis of *A. alternata*.

### Accumulation of Reactive Oxygen Species and Cell Death of *Alternaria alternata* Caused by *Streptomyces lydicus* M01 Extracts

**FIGURE 2:**
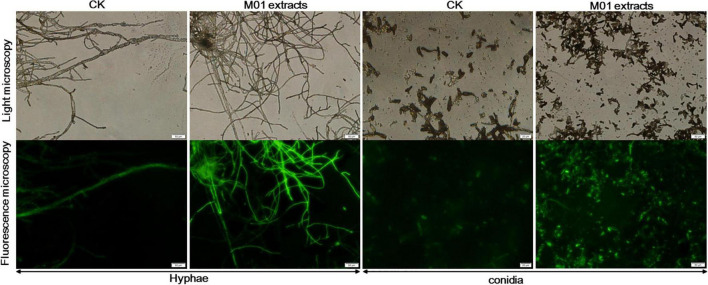
Effect of *S. lydicus* M01 extracts on ROS production by *A. alternata*.

In both eukaryotic and prokaryotic organisms, ROS are byproducts of normal metabolism. Low concentrations of ROS play important roles in physiological events, but large amounts of ROS have many damaging effects and contribute to cell death (Aguirre et al., 2005). To investigate whether the accumulation of ROS participate in the cell damage of *A. alternata* treated with *S. lydicus* M01 extracts, the oxidant-sensitive probe DCFH-DA and fluorescence microscopy were used. The staining results showed that nearly entire M01 extracts-treated mycelium showed green fluorescence and the intensity was stronger compared with control hyphae (Figure 2). And the treated conidia also had stronger fluorescence than the controls, indicating a higher ROS concentration in *S. lydicus* M01 extracts-treated hyphae and conidia.

**FIGURE 3:**
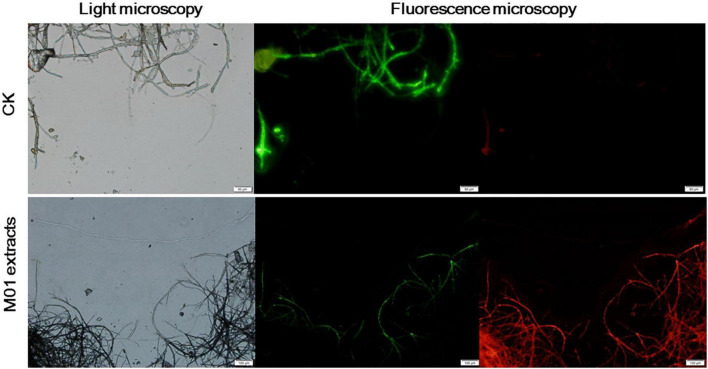
Detection of *A. alternata* viability based on fluorescein diacetate and propidium iodide staining after treatment with *S. lydicus* M01 extracts for 12 h.

To further detect whether cell death occurred, a rapid, simultaneous double-staining procedure using FDA and PI was used to analyze live and dead cells (Jones and Senft, 1985). FDA is cleaved by esterase within viable cells and liberated by the fluorescent compound fluorescein, thus viable cells retain the dye and fluoresce whereas non-viable cells do not. PI is a nucleic acid intercalating dye but excluded from live cells with intact membranes, thus viable cells show bright green fluorescence, while non-viable cells are bright red. As shown in Figure 3, untreated *A. alternata* hyphae exhibited little red fluorescence and the entire mycelium was outlined by green fluorescence. In contrast, the *S. lydicus* M01 extracts-treated mycelium had few live cells (green fluorescence) and the proportion of red fluorescence in the hyphae was significantly increased, indicating that cell death occurred when treated with *S. lydicus* M01 extracts. Combined with the microscopic and ultrastructural changes in *S. lydicus* M01 extracts-treated cells, these results indicated that the extracts of *S. lydicus* M01 could at least destroy the cell wall and membranes, thus inhibiting the growth of *A. alternata*.

### Liquid Chromatography Connected to a Quadrupole Time-of-Flight Mass Spectrometer/MS Analysis of *Streptomyces lydicus* M01 Metabolites

To characterize the antimicrobial compounds in the extracts of *S. lydicus* M01, UPLC-Q-TOF-MS was performed. A total of 123 chemical compounds were identified in the UNIFI Platform (Waters, Milford, MA, United States) based on retention time, molecular mass, and MS fragmentation (Table 1 and Supplementary Table 1). These compounds mainly consisted of fungicides, antibacterial agents, herbicides, insecticides, and plant growth regulators, such as IAA. The top 15 dominant compounds that had detector response strength above 10,000 were listed in Table 1, the remaining 108 compounds were provided in Supplementary Table 1. The response of compounds represented the relative proportion to their quantities in extracts. The listed compounds include fungicides oxadixyl, benzamorf, and coumethoxystrobin, herbicides chloreturon, S-metolachlor, fentrazamide, credazine, and paraquat, insecticide bucarpolate, isoprocarb, xylylcarb, pyraclofos, and antibacterial agent sulfamethazine. Their chemical structures were illustrated in Supplementary Figure 1. Analysis showed that the proportion of oxadixyl accounted for 35.89% in all the identified compounds, indicating that it was the dominant compound in the extracts of *S. lydicus* M01.

**TABLE 1:** Compounds identified from the extracts of *S. lydicus* M01 by UPLC-Q-TOF-MS.

Number	Predicted compounds	*t*_*R*_ (min)	Formula	*m*/*z*	Mass error (mDa)	Response	Proportion (%)
1.	Oxadixyl	4.78	C_14_H_18_N_2_O_4_	279.1334	−0.6	522,441	35.89
2.	Chloreturon	7.32	C_11_H_15_ClN_2_O_2_	243.0873	−2.2	267,954	18.41
3.	S-metolachlor	6.02	C_15_H_22_ClNO_2_	284.1389	−2.2	206,076	14.16
4.	Fentrazamide	5.85	C_16_H_20_ClN_5_O_2_	350.1379	0.1	45,308	3.11
5.	Bucarpolate	4.77	C_16_H_22_O_6_	311.1489	0	18,015	1.24
6.	Isoprocarb	3.86	C_11_H_15_NO_2_	194.117	−0.6	14,860	1.02
7.	Benzamorf	3.11	C_18_H_30_O_3_S	327.1999	1	14,160	0.97
8.	Coumethoxystrobin	5.17	C_23_H_22_O_6_	395.1487	−0.2	13,974	0.96
9.	Xylylcarb	3.13	C_10_H_13_NO_2_	180.1016	−0.3	13,883	0.95
10.	Naphthylindane-1,3-diones	5.17	C_19_H_12_O_2_	273.0883	−2.7	13,610	0.93
11.	Sulfamethazine	4.67	C_12_H_14_N_4_O_2_S	279.092	0.9	13,575	0.93
12.	Credazine	7.35	C_11_H_10_N_2_O	187.0862	−0.4	12,467	0.86
13.	Paraquat	8.96	C_12_H_14_N_2_	187.1258	2.9	12,199	0.84
14.	Pyraclofos	2.86	C_14_H_18_ClN_2_O_3_PS	361.0538	0.1	11,530	0.79
15.	Propargite	5.85	C_19_H_26_O_4_S	351.1607	−1.7	10,821	0.74

### Genome Sequencing and Metabolites Prediction of *Streptomyces lydicus* M01

**FIGURE 4:**
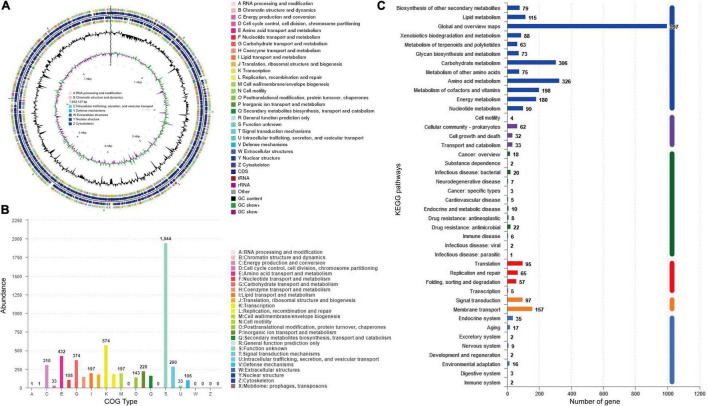
Analysis of genome structure and metabolic pathway of *S. lydicus* M01. **(A)** CGView of *S. lydicus* M01 chromosome. From outside to center, rings 1 and 4 show protein-coding genes colored by COG categories on forward/reverse strand. Rings 2 and 3 show CDS, tRNA, and rRNA on forward/reverse strand. Ring 5 represents the G + C content. Ring 6 represents GC-Skew value. **(B)** COG annotation of *S. lydicus* M01 genome. **(C)** Pathway annotation of *S. lydicus* M01 genome according to the KEGG database. The vertical axis represents the level two classification of KEGG pathway. The horizontal axis represents the number of genes annotated in this classification. Different colors of the columns represent different level one classifications of KEGG pathway.

Genome sequencing and analysis showed that the whole genome of *S. lydicus* M01 consists of a linear chromosome of 7,832,127 bp with a high G + C content of 72.04%, including 6,868 protein-coding sequences, 65 tRNA, and 21 (seven 5S, seven 16S, and seven 23S) rRNA (Figure 4A). Among a total of identified 6,868 protein-coding genes, 5,571 and 2,494 of them were annotated into COG and KEGG functional categories, respectively (Figures 4B,C). In the COG annotation, the highest ratio was the transcription, followed by amino acid transport and metabolism and carbohydrate transport and metabolism. The number of genes annotated for secondary metabolites biosynthesis, transport, and catabolism was 165. Notably, protein functions of 1,944 genes which accounted a large proportion in the genome were not annotated based on existing protein database. Based on antiSMASH software analysis of the *S. lydicus* M01 genome, 24 biosynthetic gene clusters coding for secondary metabolites were detected, mainly included five NRPS gene clusters, one type I PKS gene cluster, one type II PKS gene cluster, one type III PKS gene cluster, two lasso peptide gene clusters, one lanthipeptide gene cluster, one bacteriocin gene cluster, four terpene gene clusters, two siderophore gene clusters, one ectoine gene cluster, two butyrolactone gene clusters, and other gene clusters (Supplementary Table 2). By alignment of GenBank, the eight gene clusters that showed a similarity above 69% were annotated in Table 2, including four gene clusters with 100% similarity. The structures of these predicted chemicals were demonstrated in Supplementary Figure 2. Function predictions revealed that five gene clusters were involved in the biosynthesis of antimicrobial-associated metabolites, including citrulassin D, streptolydigin, mannopeptimycin, desferrioxamine E, and SapB (Table 2). In addition, other unmatched gene clusters, including NRPS, type I PKS, type III PKS, RiPP-like, and lasso peptide gene cluster, were also found in the genome, which might be involved in the biosynthesis of other antimicrobial metabolites.

**TABLE 2:** Prediction and functional annotation of secondary metabolites from *S. lydicus* M01 genome.

Cluster ID	Type	Gene position (bp)	Predicted compounds	Similarity (%)	Functions	References
Cluster1	Lasso peptide	214,129–234,237	Citrulassin D	100	Antimicrobial activity	Tietz et al., 2017
Cluster4	Lanthipeptide-class-iii	718,718–741,331	SapB	100	Spore-associated peptide	Dischinger et al., 2014
Cluster6	T2PKS	1,107,620–1,180,136	Spore pigment	83	spore pigment	Omura et al., 2001
Cluster7	NRPS, T1PKS, terpene	1,491,890–1,602,667	Streptolydigin	97	Antibiotic, antiviral, antifungal	Tuske et al., 2005
Cluster8	Siderophore	2,057,190–2,065,916	Desferrioxamine E	100	Chelating agent, antibacterial	Ishida et al., 2011
Cluster9	Ectoine	2,144,362–2,154,779	Ectoine	100	Osmotic stress compatible solute	Prabhu et al., 2004
Cluster14	NRPS	4,721,858–4,780,039	Mannopeptimycin	81	Antibiotics	Magarvey et al., 2006
Cluster20	Terpene	7,124,194–7,149,633	Hopene	69	Cell membrane associated	Bentley et al., 2002

## Discussion

Excessive use of chemical fertilizers had adverse effects on the environment, causing pollution of soil, water, and air. The development of sustainable agriculture requires biological based fertilizers composed of living microorganisms. The plant-associated microbes that were beneficial for plant growth and disease suppression were used in the bio-based fertilizers or as biocontrol agents. Species from the *Streptomyces* genus are typical representatives of plant growth-promoting rhizobacteria, they colonize plant roots, fight against pathogens, synthesize extracellular proteins, and produce a wide range of bioactive compounds. For example, an endophytic actinomycete, *Streptomyces* sp. MBR-52 was found to accelerate the emergence and elongation of plant adventitious roots (Meguro et al., 2006). Three endophytic actinomycetes strains recovered from the root tissues of *Azadirachta indica* A. Juss. (Meliaceae) prolifically produce IAA and siderophores that play vital roles in the promotion of plant growth (Verma et al., 2011). *Streptomyces* sp. MTN14 was reported to enhance the biomass yield of *Withania somnifera* (Singh et al., 2016). Importantly, the metabolites of *Streptomyces* are diversified in biological activities and functions, such as antifungal, insecticidal, and antibacterial activities, makes them the promising candidates to be developed as efficient biocontrol agents (Rey and Dumas, 2017). In recent studies, we isolated a new stain *S. lydicus* M01 that exhibited outstanding plant disease suppression and growth promotion properties on cucumber plants (Wang et al., 2020). To further investigate its antimicrobial mechanism, physiological changes of plant pathogen *A. alternata*, secondary metabolite profiles, and genome sequencing of *S. lydicus* M01 were analyzed.

The morphological changes observed using scanning electron microscopy and transmission electron microscopy showed that the *S. lydicus* M01 extracts disrupted the plasma membranes of *A. alternata* hyphae and conidia and caused cytoplasmic heterogeneity and organelles disintegration. These results were similar to that observed in lipopeptides, for example, lipopeptide surfactin induced lysis of lipid membranes and leakage (Heerklotz and Seelig, 2007). Iturin and bacillomycin D cause cytoplasm extravasation of *Verticillium dahliae* and *Aspergillus flavus*, respectively (Gong et al., 2014; Han et al., 2015). However, cell swelling and breakage of *A. alternata* cell walls were not observed in our study. This result differed from findings observed in bacterial lipopeptide iturins, possibly due to the different antifungal components in the extracts of *S. lydicus* M01. The organelles disintegration observed may suggest that the antifungal components in the *S. lydicus* M01 extract could enter the cells and affect organelles and intracellular proteins in the cytoplasm.

Increased accumulation of ROS is harmful to fungal cells (Mesa-Arango Ana et al., 2014; Thangamani et al., 2017). Recent studies showed that lipopeptide induced cell death in part by generating ROS (Qi et al., 2010; Cao et al., 2011). In our experiments, high concentrations of ROS were also detected in *S. lydicus* M01 extracts-treated hyphae and conidia, suggesting that ROS generation may contribute to the antifungal effects of the *S. lydicus* M01 extracts. In the double-staining of *S. lydicus* M01 extracts-treated hyphae and conidia, significant cell death was observed. This result also demonstrated that the plasma membranes were disrupted since PI was not membrane-permeable (Lecoeur, 2002). Taken together, our results and previous results suggested that the extracts of *S. lydicus* M01 caused severe damage to the plasma membranes and organelles disintegration of hyphae and conidia, resulting in cell death of *A. alternata*.

*Streptomyces* are known to be rich in secondary metabolites; they produce over two-thirds of the clinically useful antibiotics of natural origin (Watve et al., 2001; Bibb, 2013). To further investigate the antimicrobial mechanism of *S. lydicus* M01, UPLC-Q-TOF-MS was used to analyze the components in the extracts of *S. lydicus* M01. An excess of 120 chemical compounds were identified, mainly consisted of fungicides, antibacterial agents, herbicides, insecticides, and plant growth regulators, such as IAA. The dominant compound which account for 35.89% in all the identified components was oxadixyl. Oxadixyl [2-methoxy-N-(2-oxo-1,3-oxazolidin-3-yl)-acet-2′,6′-xylidide] is a systemic fungicide used to treat seeds of a variety of food crops, including control of downy mildew, damping-off (*Phytophthora* and *Pythium*), and seed rot (*Pythium*) (Jiang and Grossmann, 1991; Samoucha et al., 1993; Heiberg, 1998). Benzamorf is also a fungicide and coumethoxystrobin is a novel Chinese strobilurin fungicide used in agriculture (Lewis et al., 2016). Chloreturon, S-metolachlor, fentrazamide, credazine, and paraquat are selective and systemic herbicides used on corn, soybeans, potatoes, tomatoes, and rice (Forouzesh et al., 2015). The remaining bucarpolate, isoprocarb, xylylcarb, and pyraclofos are insecticide or insecticide synergists (Sun et al., 2012). In addition, sulfamethazine is an antibacterial agent that are widely used in human and veterinary medicine (Haller et al., 2002). These metabolites detected were all small molecules with low molecular weight, their antimicrobial mechanisms are probably different from large biomacromolecule, such as lipopeptides. These small molecules could inhibit enzymes that are involved in cell metabolism or they can disrupt protein synthesis and membrane structure, thus explaining the morphological changes of pathogen cells observed in this study. However, it was reported that lytic enzymes, such as chitinase, may participate in the antifungal activities of *S. lydicus* and other *Streptomyces*; the detailed mechanism of antifungal enzymes were not investigated (Mahadevan and Crawford, 1997; Mukherjee and Sen, 2006).

Genome sequencing provides the potential to discover secondary metabolites produced by *Streptomyces* (Bachmann et al., 2014; Kjærbølling et al., 2018; Skinnider et al., 2020). In this study, genome sequencing of *S. lydicus* M01 revealed 6,868 protein-coding sequences and 24 potential biosynthetic gene clusters coding for secondary metabolites. The NRPS gene clusters, PKS gene clusters, lasso peptide gene clusters, lanthipeptide gene cluster, and terpene gene clusters discovered in *S. lydicus* M01 genome are putatively involved in biosynthesis of antimicrobial-associated metabolites. Based on alignment with repository of known biosynthetic gene clusters, the predicted products were citrulassin D, streptolydigin, mannopeptimycin, and SapB. Citrulassin D belongs to lasso peptides that exhibited antibacterial activity against a panel of bacteria, including pathogen *Listeria monocytogenes* and *Klebsiella pneumoniae* (Tietz et al., 2017). The streptolydigin gene cluster showed 97% similarity with the corresponding genes in other strains of *S. lydicus* (Table 2). Streptolydigin is an antibiotic that inhibits nucleic acid chain elongation by binding to RNA polymerase, thus inhibiting RNA synthesis (Tuske et al., 2005). It has antibacterial activity against a number of bacteria but not eukaryotic microbes. Mannopeptimycin which is produced by *Streptomyces* is a new class of lipoglycopeptide antibiotics that acts by affecting cell wall biosynthesis (Magarvey et al., 2006). SapB is a lantibiotic-like peptide but belongs to class III lanthipeptides and lacks antimicrobial activity (Dischinger et al., 2014). It performed morphogenetic and signaling functions for the filamentous bacterium *Streptomyces* (Kodani et al., 2004). In addition, the siderophore cluster shared 100% similarities with desferrioxamine E which is widely produced by *Streptomyces* and related bacteria. Desferrioxamine E was reported to inhibit biofilm formation of *Mycobacterium* species and thus exhibited antimicrobial activity (Ishida et al., 2011).

Interestingly, these four predicted antibacterial compounds were not detected by UPLC-Q-TOF-MS in the analysis of *S. lydicus* M01 extracts. This might be explained by the different extraction and identification methods. It was reported that many of these bio-informatically discovered secondary metabolism gene clusters were silent or expressed at low levels under standard laboratory conditions (Brakhage and Schroeckh, 2011). The expression of these gene clusters is controlled by biological signals and regulatory networks governed by stresses found in the bacteria’s natural habitat (Craney et al., 2013). Therefore, the secondary metabolites detected could only be a small fraction of the whole repository. Taken together, we propose that the compounds detected in the extracts of *S. lydicus* M01 and bio-informatically predicted secondary metabolites could altogether contribute to the antifungal activity of *S. lydicus* M01. Further investigation of the secondary metabolites and genome mining of *S. lydicus* M01 should help in understanding its antimicrobial mechanism.

## Conclusion

In conclusion, the antimicrobial mechanism of biocontrol agent *S. lydicus* M01 against leaf spot pathogen *A. alternata* was investigated. The results revealed that the extracts of *S. lydicus* M01 caused damage of plasma membranes and cytoplasm extravasation of *A. alternata*. The accumulation of ROS contributed to the antifungal effects of the *S. lydicus* M01 extracts. Analysis of secondary metabolite profiles using ultra-high-performance liquid chromatography connected to a quadrupole time-of-flight mass spectrometer (UPLC-Q-TOF-MS) revealed an excess of 120 metabolites, mainly consisting of fungicides, antibacterial agents, herbicides, insecticides, and plant growth regulators, such as IAA. On the other hand, the complete genome sequencing revealed a number of key function gene clusters that contribute to the biosynthesis of active secondary metabolites. These results provided insights for a comprehensive understanding of the biocontrol agent *S. lydicus* and may pave the way to the use of their metabolites in agriculture.

## Data Availability Statement

The datasets presented in this study can be found in online repositories. The names of the repository/repositories and accession number(s) can be found at: https://www.ncbi.nlm.nih.gov/, PRJNA774437.

## Author Contributions

MW contributed to conceptualization, methodology, investigation, validation, formal analysis, visualization, writing—original draft, and funding acquisition. JL and WC contributed to conceptualization, methodology, and investigation. JZ contributed to conceptualization, supervision, and writing, reviewing, and editing the manuscript. All authors contributed to the article and approved the submitted version.

## Conflict of Interest

The authors declare that the research was conducted in the absence of any commercial or financial relationships that could be construed as a potential conflict of interest.

## Publisher’s Note

All claims expressed in this article are solely those of the authors and do not necessarily represent those of their affiliated organizations, or those of the publisher, the editors and the reviewers. Any product that may be evaluated in this article, or claim that may be made by its manufacturer, is not guaranteed or endorsed by the publisher.

## Footnotes

1
www.majorbio.com
