# Supplementary material for: Antimicrobial Mechanism and Secondary Metabolite Profiles of Biocontrol Agent Streptomyces lydicus M01 Based on Ultra-High-Performance Liquid Chromatography Connected to a Quadrupole Time-of-Flight Mass Spectrometer Analysis and Genome Sequencing
Source: Front Microbiol. 2022 May 31;13:908879. doi: 10.3389/fmicb.2022.908879 (PMC9194905; doi:10.3389/fmicb.2022.908879)
Supplement: Supplementary file 1 [file Data_Sheet_1.docx]

Supplementary Material

# Supplementary Figures and Tables

## Supplementary Figures


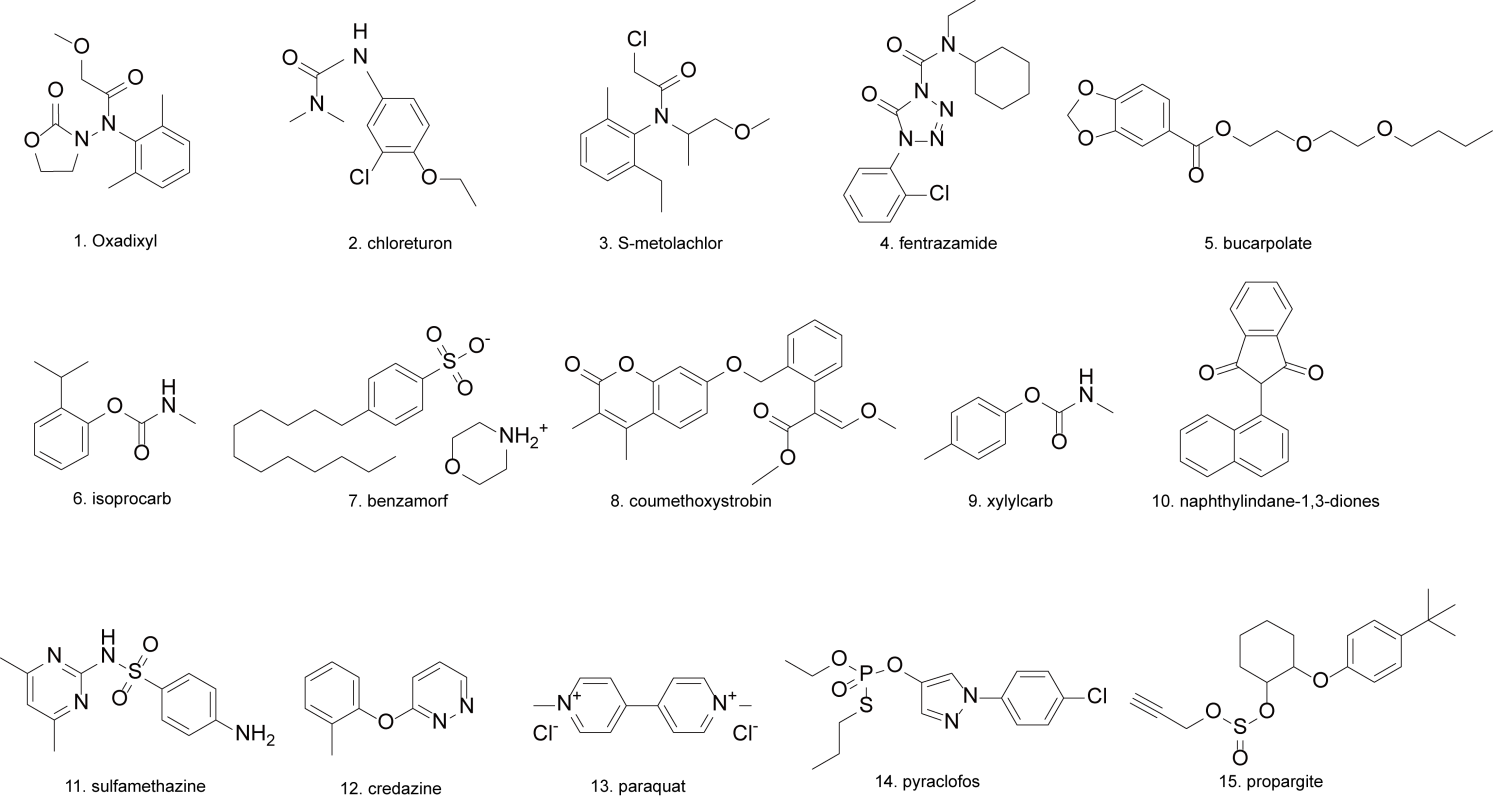


**Supplementary Figure 1.** Chemical structures of the identiﬁed compounds from the extracts of *S. lydicus* M01.


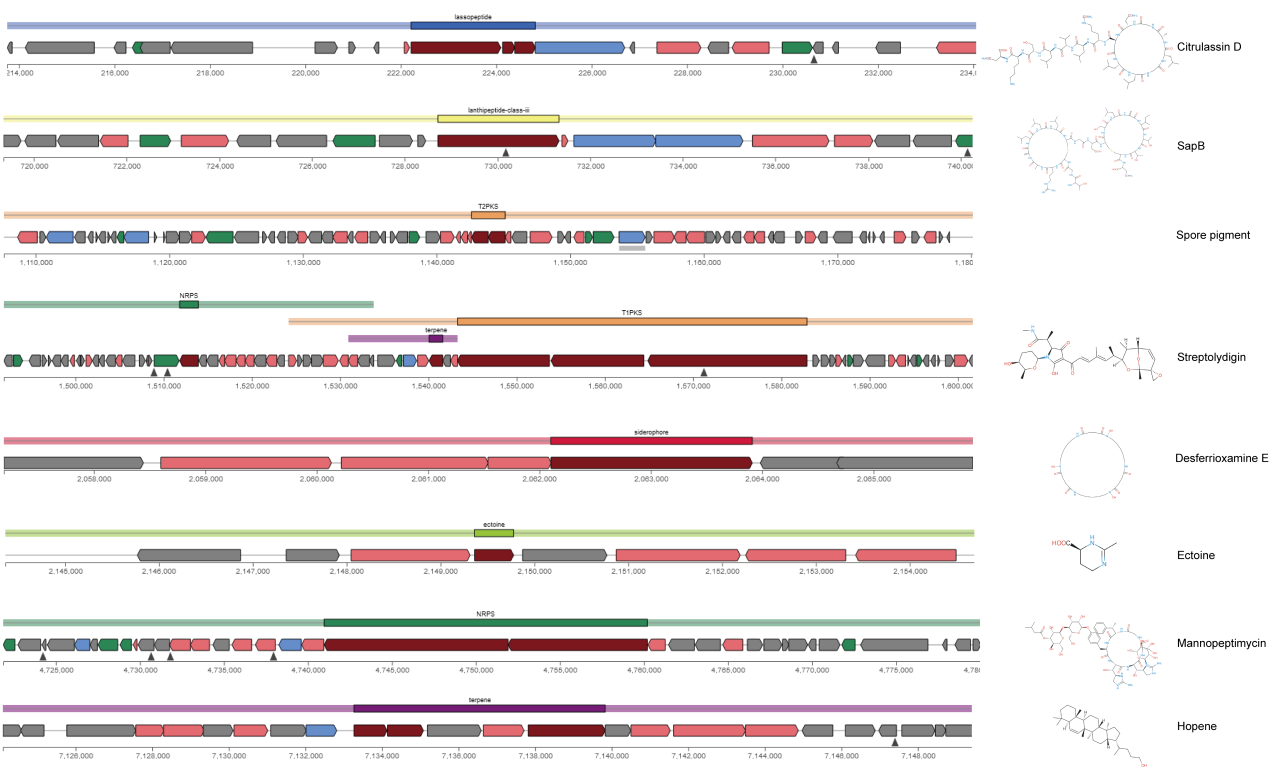


**Supplementary Figure 2.** Analysis of gene clusters related to the biosynthesis of secondary metabolites using the online antiSMASH v6.0.1 software.

## Supplementary Tables

**Table S1** Compounds identified from the extracts of *S. lydicus* M01 by UPLC-Q-TOF-MS

| number | Predicted compounds | *t*_R_  (min) | formula | m/z | mass  error  (mDa) | Response | Proportion (%) |
| --- | --- | --- | --- | --- | --- | --- | --- |
| 1 | sethoxydim | 3.51 | C_17_H_29_NO_3_S | 328.1953 | 1.2 | 9544 | 0.66 |
| 2 | desoxymetasone | 5.08 | C_22_H_29_FO_4_ | 377.213 | 0.7 | 8663 | 0.60 |
| 3 | dichlorprop-isoctyl | 3.71 | C_17_H_24_C_l2_O_3_ | 347.1192 | 1.7 | 8187 | 0.56 |
| 4 | dibutyl phthalate | 1.95 | C_16_H_22_O_4_ | 279.1579 | -1.1 | 7844 | 0.54 |
| 5 | azobenzene | 4.96 | C_12_H_10_N_2_ | 183.0911 | -0.5 | 7061 | 0.49 |
| 6 | carbofuran | 0.96 | C_12_H_15_NO_3_ | 222.1119 | -0.6 | 6951 | 0.48 |
| 7 | imazamethabenz | 6.76 | C_15_H_18_N_2_O_3_ | 275.1385 | -0.5 | 6791 | 0.47 |
| 8 | clotrimazole | 4.08 | C_22_H_17_ClN_2_ | 345.1179 | 2.6 | 6707 | 0.46 |
| 9 | flurenol-butyl | 6.02 | C_18_H_18_O_3_ | 283.1315 | -1.3 | 6247 | 0.43 |
| 10 | IAA | 2.85 | C_10_H_9_NO_2_ | 176.0702 | -0.4 | 6098 | 0.42 |
| 11 | flunisolide | 5.28 | C_24_H_31_FO_6_ | 435.2187 | 1 | 6087 | 0.42 |
| 12 | dichlorprop-P-2-ethylhexyl | 3.85 | C_17_H_24_Cl_2_O_3_ | 347.1184 | 0.9 | 6056 | 0.42 |
| 13 | pirimicarb | 2.45 | C_11_H_18_N_4_O_2_ | 239.148 | -2.3 | 5786 | 0.40 |
| 14 | ribavirin | 4.51 | C_8_H_12_N_4_O_5_ | 245.09 | 1.9 | 5768 | 0.40 |
| 15 | benzamacril | 5.23 | C_12_H_12_N_2_O_2_ | 217.098 | 0.9 | 5488 | 0.38 |
| 16 | karbutilate | 7.33 | C_14_H_21_N_3_O_3_ | 280.1656 | 0 | 5313 | 0.36 |
| 17 | sulfathiazole | 6.24 | C_9_H_9_N_3_O_2_S_2_ | 256.0201 | -0.8 | 5213 | 0.36 |
| 18 | medlure | 2.86 | C_12_H_21_ClO_2_ | 233.1278 | -2.5 | 5110 | 0.35 |
| 19 | dexamethasone | 4.26 | C_22_H_29_FO_5_ | 393.2065 | -0.7 | 4950 | 0.34 |
| 20 | pethoxamid | 6.27 | C_16_H_22_ClNO_2_ | 296.1417 | 0.5 | 4342 | 0.30 |
| 21 | aminocarb | 3.85 | C_11_H_16_N_2_O_2_ | 209.1277 | -0.7 | 4299 | 0.30 |
| 22 | mazidox | 3.47 | C_4_H_12_N_5_OP | 178.0859 | 0.7 | 4017 | 0.28 |
| 23 | mefluidide | 3.85 | C_11_H_13_F_3_N_2_O_3_S | 311.0683 | 1.1 | 4007 | 0.28 |
| 24 | fluenetil | 3.39 | C_16_H_15_FO_2_ | 259.1141 | 1.2 | 3977 | 0.27 |
| 25 | propamidine | 6.28 | C_17_H_20_N_4_O_2_ | 313.1647 | -1.2 | 3953 | 0.27 |
| 26 | triamcinolone acetonide | 5.08 | C_24_H_31_FO_6_ | 435.2181 | 0.4 | 3674 | 0.25 |
| 27 | tribenuron | 3.76 | C_14_H_15_N_5_O_6_S | 382.0819 | 0.3 | 3519 | 0.24 |
| 28 | quinazamid | 1.85 | C_7_H_7_N_3_O_2_ | 166.061 | -0.1 | 3271 | 0.22 |
| 29 | acetochlor | 6.5 | C_14_H_20_ClNO_2_ | 270.1231 | -2.5 | 3184 | 0.22 |
| 30 | cisanilide | 8.1 | C_13_H_18_N_2_O | 219.1477 | -1.5 | 3041 | 0.21 |
| 31 | flumequine | 5.15 | C_14_H_12_FNO_3_ | 262.0884 | 1 | 2753 | 0.19 |
| 32 | sebuthylazin-desethyl | 4.16 | C_7_H_12_ClN_5_ | 202.0829 | -2.5 | 2702 | 0.19 |
| 33 | ancymidol | 6.76 | C_15_H_16_N_2_O_2_ | 257.1278 | -0.7 | 2613 | 0.18 |
| 34 | thiabendazole | 3.64 | C_10_H_7_N_3_S | 202.0459 | 2.5 | 2502 | 0.17 |
| 35 | bilanafos | 3.3 | C_11_H_22_N_3_O_6_P | 324.1292 | -2.7 | 2422 | 0.17 |
| 36 | diphacinone | 3.07 | C_23_H_16_O_3_ | 341.1196 | 2.4 | 2203 | 0.15 |
| 37 | curcumenol | 0.73 | C_15_H_22_O_2_ | 235.168 | -1.3 | 2177 | 0.15 |
| 38 | carboxazole | 3.04 | C_9_H_14_N_2_O_3_ | 199.1069 | -0.8 | 1889 | 0.13 |
| 39 | etaconazole I | 4.21 | C_14_H_15_Cl_2_N_3_O_2_ | 328.0634 | 2 | 1832 | 0.13 |
| 40 | mecoprop-dimethylammonium | 2.6 | C_12_H_18_ClNO_3_ | 260.102 | -2.8 | 1813 | 0.12 |
| 41 | trifop-methyl | 4.17 | C_17_H_15_F_3_O_4_ | 341.1014 | 1.8 | 1779 | 0.12 |
| 42 | propoxur | 3.84 | C_11_H_15_NO_3_ | 210.1108 | -1.7 | 1767 | 0.12 |
| 43 | IBA | 3.53 | C_12_H_13_NO_2_ | 204.1011 | -0.8 | 1758 | 0.12 |
| 44 | imazamethabenz-methyl | 5.25 | C_16_H_20_N_2_O_3_ | 289.155 | 0.3 | 1676 | 0.12 |
| 45 | fuberidazole | 5.44 | C_11_H_8_N_2_O | 185.0703 | -0.7 | 1654 | 0.11 |
| 46 | metominostrobin(E-) | 4.86 | C_16_H_16_N_2_O_3_ | 285.122 | -1.4 | 1645 | 0.11 |
| 47 | atrazine | 4.98 | C_8_H_14_ClN_5_ | 216.1009 | -0.2 | 1622 | 0.11 |
| 48 | zolaprofos | 3.04 | C_10_H_18_NO_3_PS_2_ | 296.0528 | -1 | 1615 | 0.11 |
| 49 | phenazine oxide | 6.72 | C_12_H_8_N_2_O | 197.0717 | 0.8 | 1607 | 0.11 |
| 50 | imazaquin | 7.61 | C_17_H_17_N_3_O_3_ | 312.1332 | -1.1 | 1604 | 0.11 |
| 51 | imazapic | 2.71 | C_14_H_17_N_3_O_3_ | 276.1333 | -1 | 1577 | 0.11 |
| 52 | fluoridamid | 2.91 | C_10_H_11_F_3_N_2_O_3_S | 297.0518 | 0.3 | 1569 | 0.11 |
| 53 | thicrofos | 2.86 | C_13_H_18_ClO_3_PS_2_ | 353.0175 | -2.1 | 1563 | 0.11 |
| 54 | quinacetol | 3.06 | C_11_H_9_NO_2_ | 188.0734 | 2.8 | 1551 | 0.11 |
| 55 | karetazan | 2.91 | C_15_H_14_ClNO_3_ | 292.0711 | -2.4 | 1495 | 0.10 |
| 56 | flunixin | 3.8 | C_14_H_11_F_3_N_2_O_2_ | 297.0821 | -2.5 | 1442 | 0.10 |
| 57 | etacelasil | 4.72 | C_11_H_25_ClO_6_Si | 317.117 | -1.1 | 1428 | 0.10 |
| 58 | oxadiazon | 2.86 | C_15_H_18_Cl_2_N_2_O_3_ | 345.0752 | -1.5 | 1358 | 0.09 |
| 59 | benomyl | 3.6 | C_14_H_18_N_4_O_3_ | 291.1435 | -1.7 | 1355 | 0.09 |
| 60 | TEPP | 6.44 | C_8_H_20_O_7_P_2_ | 291.0779 | 2.2 | 1333 | 0.09 |
| 61 | aviglycine hydrochloride | 4.11 | C_6_H_13_ClN_2_O_3_ | 197.0676 | -1.1 | 1301 | 0.09 |
| 62 | sulfaquinoxaline | 0.4 | C_14_H_12_N_4_O_2_S | 301.0743 | -1.1 | 1292 | 0.09 |
| 63 | EPN | 3.38 | C_14_H_14_NO_4_PS | 324.0429 | -2.5 | 1287 | 0.09 |
| 64 | ciobutide | 3.27 | C_11_H_12_N_2_O | 189.1016 | -0.7 | 1261 | 0.09 |
| 65 | tulobuterol | 3.01 | C_12_H_18_ClNO | 228.1128 | -2.1 | 1253 | 0.09 |
| 66 | deoxynivalenol | 4.14 | C_15_H_20_O_6_ | 297.1322 | -1.1 | 1246 | 0.09 |
| 67 | azaconazole | 2.51 | C_12_H_11_Cl_2_N_3_O_2_ | 300.0295 | -0.6 | 1244 | 0.09 |
| 68 | fenoprop-isoctyl | 4.22 | C_17_H_23_Cl_3_O_3_ | 381.0783 | -0.2 | 1189 | 0.08 |
| 69 | griseofulvin | 5.16 | C_17_H_17_ClO_6_ | 353.0799 | 1.3 | 1164 | 0.08 |
| 70 | imidacloprid | 3.78 | C_9_H_10_ClN_5_O_2_ | 256.0571 | -2.5 | 1160 | 0.08 |
| 71 | fluazifop-P | 3.27 | C_15_H_12_F_3_NO_4_ | 328.079 | -0.1 | 1117 | 0.08 |
| 72 | acetophos | 3.38 | C_8_H_17_O_5_PS | 257.0608 | 0.1 | 1116 | 0.08 |
| 73 | chlorfluren | 3.64 | C_14_H_9_ClO_2_ | 245.0363 | -0.1 | 1096 | 0.08 |
| 74 | 2,4-D-2-ethylhexyl | 2.91 | C_16_H_22_Cl_2_O_3_ | 333.0999 | -1.9 | 1083 | 0.07 |
| 75 | dinitramine | 3.46 | C_11_H_13_F_3_N_4_O_4_ | 323.0991 | 3 | 1071 | 0.07 |
| 76 | haloxydine | 0.51 | C_5_HCl_2_F_2_NO | 199.9496 | 2 | 1066 | 0.07 |
| 77 | dinotefuran | 2.98 | C_7_H_14_N_4_O_3_ | 203.1161 | 2.2 | 1045 | 0.07 |
| 78 | pyributicarb | 1.3 | C_18_H_22_N_2_O_2_S | 331.1491 | 1.6 | 1029 | 0.07 |
| 79 | dimefuron | 3.11 | C_15_H_19_ClN_4_O_3_ | 339.1206 | -1.2 | 982 | 0.07 |
| 80 | bensulfuron | 3.94 | C_15_H_16_N_4_O_7_S | 397.0792 | -2 | 970 | 0.07 |
| 81 | propiconazole I | 6.79 | C_15_H_17_Cl_2_N_3_O_2_ | 342.0774 | 0.4 | 969 | 0.07 |
| 82 | lenacil | 3.62 | C_13_H_18_N_2_O_2_ | 235.1413 | -2.8 | 954 | 0.07 |
| 83 | atrazine-desisopropyl | 3.26 | C_5_H_8_ClN_5_ | 174.0541 | 0 | 950 | 0.07 |
| 84 | pyrasulfotole | 3.62 | C_14_H_13_F_3_N_2_O_4_S | 363.0608 | -1.3 | 943 | 0.06 |
| 85 | nitrothal-isopropyl | 3.46 | C_14_H_17_NO_6_ | 296.1143 | 1.5 | 933 | 0.06 |
| 86 | xylachlor | 3.36 | C_13_H_18_ClNO | 240.1122 | -2.8 | 932 | 0.06 |
| 87 | quinothion | 2.91 | C_14_H_18_NO_3_PS | 312.0838 | 2 | 921 | 0.06 |
| 88 | mefenpyr-diethyl | 2.86 | C_16_H_18_Cl_2_N_2_O_4_ | 373.0711 | -0.5 | 919 | 0.06 |
| 89 | difenoxuron | 0.75 | C_16_H_18_N_2_O_3_ | 287.1383 | -0.7 | 897 | 0.06 |
| 90 | benquinox | 3.9 | C_13_H_11_N_3_O_2_ | 242.0913 | -1.1 | 895 | 0.06 |
| 91 | phenobenzuron | 2.86 | C_16_H_14_Cl_2_N_2_O_2_ | 337.0478 | -2.7 | 884 | 0.06 |
| 92 | malaoxon | 4.17 | C_10_H_19_O_7_PS | 315.0647 | -1.5 | 880 | 0.06 |
| 93 | amiton oxalate | 4.17 | C_10_H_24_NO_3_PS.C_2_H_2_O_4_ | 360.125 | 0.9 | 871 | 0.06 |
| 94 | fluazifop | 3.46 | C_15_H_12_F_3_NO_4_ | 328.0786 | -0.6 | 871 | 0.06 |
| 95 | diphenylamine | 1.22 | C_12_H_11_N | 170.0954 | -1 | 869 | 0.06 |
| 96 | thiophanate | 3.85 | C_14_H_18_N_4_O_4_S_2_ | 371.0814 | -2.8 | 831 | 0.06 |
| 97 | tetcyclacis | 4.65 | C_13_H_12_ClN_5_ | 274.0864 | 1 | 814 | 0.06 |
| 98 | DMST | 3.98 | C_9_H_14_N_2_O_2_S | 215.0831 | -1.8 | 776 | 0.05 |
| 99 | triflupromazine | 1.3 | C_18_H_19_F_3_N_2_S | 353.13 | 0.6 | 766 | 0.05 |
| 100 | rotenone | 4.65 | C_23_H_22_O_6_ | 395.148 | -0.9 | 754 | 0.05 |
| 101 | diniconazole-M | 3.27 | C_15_H_17_Cl_2_N_3_O | 326.0792 | -3 | 752 | 0.05 |
| 102 | trifop | 2.91 | C_16_H_13_F_3_O_4_ | 327.0862 | 2.3 | 644 | 0.04 |
| 103 | fenuron | 2.99 | C_9_H_12_N_2_O | 165.1015 | -0.7 | 641 | 0.04 |
| 104 | penconazole | 3.27 | C_13_H_15_Cl_2_N_3_ | 284.0695 | -2.1 | 635 | 0.04 |
| 105 | shuangjiaancaolin | 4.17 | C_12_H_19_N_2_O_4_PS | 319.0893 | 1.7 | 606 | 0.04 |
| 106 | dithiopyr | 3.76 | C_15_H_16_F_5_NO_2_S_2_ | 402.0604 | -1.1 | 597 | 0.04 |
| 107 | busulfan | 0.55 | C_6_H_14_O_6_S_2_ | 247.0293 | -1.1 | 477 | 0.03 |
| 108 | mecarbam | 4.23 | C_10_H_20_NO_5_PS_2_ | 330.0565 | -2.8 | 453 | 0.03 |

**Table S2** Gene clusters coding for secondary metabolites predicted in *S. lydicus* M01 genome.

| Cluster Type | Cluster Number | Gene Number |
| --- | --- | --- |
| NRPS | 5 | 258 |
| type I PKS | 1 | 37 |
| type II PKS | 1 | 74 |
| type III PKS | 1 | 54 |
| lassopeptide | 2 | 37 |
| lanthipeptide | 1 | 19 |
| bacteriocin | 1 | 6 |
| terpene | 4 | 78 |
| siderophore | 2 | 16 |
| ectoine | 1 | 8 |
| butyrolactone | 2 | 18 |
